# A Systematic Review of Treatment for Children with Autism Spectrum Disorder: The Sensory Processing and Sensory Integration Approach

**DOI:** 10.3390/children11101222

**Published:** 2024-10-09

**Authors:** Jonathan Camino-Alarcón, Maria Auxiliadora Robles-Bello, Nieves Valencia-Naranjo, Aziz Sarhani-Robles

**Affiliations:** 1 Department of Psychology, University of Jaen, 23071 Jaen, Spain; jvca0001@red.ujaen.es (J.C.-A.); nnaranjo@ujaen.es (N.V.-N.); 2 Medicine Faculty, University of Granada, 18016 Granada, Spain; asarhanir@correo.ugr.es

**Keywords:** sensory integration, assessment, treatment, autism spectrum disorder, systematic review

## Abstract

Background/Objectives: The prevalence of the diagnosis of autism spectrum disorder (ASD) has been increasing globally, necessitating updates to the Diagnostic and Statistical Manual of Mental Disorders with respect to ASD diagnosis. It is now recognised that ASD is related to sensory processing disorder, and sensory integration is considered a suitable intervention for treating children diagnosed with ASD. Methods: This paper provides a systematic review on a timeline from 2013 to 2023, based on the PRISMA model. Evidence was sought in the academic search engines Pubmed, Scielo, Eric, Dialnet, Springer, Base Search and Google Scholar, which produced 16 articles according to the inclusion criteria. Results: According to the results of this review, intervention with sensory integration in infants with ASD meets the criteria to be considered an evidence-based practice. The studies reviewed focused mainly on clinical settings and, therefore, we highlight the urgent need for further research to evaluate the effectiveness of sensory integration interventions in naturalistic settings such as homes and schools. Conclusions: This will help to obtain more representative data on how these interventions affect the daily lives of children with ASD.

## 1. Introduction

Today, the term autism spectrum disorder (ASD) is used to describe a clinically heterogeneous group of neurodevelopmental disorders that share core behavioural features that affect social communication and include stereotypic, restrictive and repetitive behaviour patterns and interests. This term refers to several conditions as idiopathic forms, including autism, Asperger’s syndrome, pervasive developmental disorders not otherwise specified, childhood disintegrative disorder and Rett syndrome. With the establishment of the Diagnostic and Statistical Manual of Mental Disorders (DSM-5), these previously separate diagnoses have been grouped under a unifying umbrella called ASD. ASD is also associated with many comorbidities, which can be physiological and psychological [1]. These changes were introduced to create a simpler and clearer system for identifying and diagnosing ASD, based on the number of specific symptoms or deficits needed to meet the diagnostic criteria [2] (Peters and Matson, 2020).

However, ASD research is heavily biased towards high-income Western countries. In most low- and middle-income countries, where the majority of people with ASD worldwide live, culturally appropriate ASD screening and diagnostic tools are lacking. Although the domains that define ASD are found at a global level, subtle differences in specific manifestations of symptoms, at the subdomain or behavioural level, may exist in different ethnic groups. Therefore, cultural differences in the expression of ASD should be taken into account, e.g., in Chinese culture, eye contact with authority is traditionally considered shameful, whereas in some African or Latin cultures, it is considered disrespectful. In these cultures, children who do not make eye contact with parents, teachers, or physicians may be perceived as compliant rather than exhibiting atypical autistic behaviours, so the eye contact abnormalities described in the DSM-5 may be more subtle and difficult to identify. On the other hand, in India, there is a common local cultural belief that boys start speaking later than girls, while in China, a language delay is often interpreted as a good sign, so even when a language delay is recognised, cultural beliefs may influence the perception that this delay may influence when help is sought. Imaginative play is another example of an autistic symptom where differences in cultural norms may be related to general population samples from India, Kenya and Mexico, where less than half of the children demonstrated imaginative play. Thus, a lack of imaginative play may be a more prominent symptom of ASD in high-income Western countries than in other cultural contexts [3] (De Leeuw et al., 2020).

The prevalence of ASD has been increasing, and one explanation for this is the development and use of common diagnostic tools validated by practitioners and researchers worldwide [4]. The Centers for Disease Control and Prevention reported in 2018 that 1 in 59 children in the United States is diagnosed with ASD, with more than 90% estimated to show features of sensory disorganisation [5]. In contrast, the prevalence of ASD in European countries from 2008 to 2018 was 1/172 in Denmark, 1/125 in Norway, 1/64 in the UK, 1/806 in Portugal, 1/44, 1/175 in the Netherlands, 1/87 in Italy, 1/166 in Germany and 1/64 (3–4 years) or 1/100 (10–11 years) in Spain [4]. The way states collect and report data on ASD can vary widely. For this reason, a high prevalence of ASD can be seen in the United States due to its Autism Spectrum Disorders Surveillance System, which can collect data in greater detail and more comprehensively [5].

The fifth edition of the DSM (APA, 2013) [6,7] established new diagnostic criteria for ASD with some significant changes (see Table 1), such as the symptoms required for diagnosis being reduced from 3 to 2, the strict restrictions recommended for age of onset being removed and symptoms such as sensory hyper- or hypo-reactivity being added [6]. Criterion A of the DSM is as follows: (A) ongoing deficiencies in social communication and social interaction in different contexts, such as (A1) deficiencies in socioemotional reciprocity; (A2) deficiencies in non-verbal communicative behaviours used in social interaction; (A3) deficiencies in the development, maintenance and understanding of relationships [8].

According to the deficits specified by the DSM-5 with respect to communication and social interaction, infants with ASD characteristics may manifest certain behaviours, such as failure to take turns, difficulty in maintaining adequate joint attention and complications in communication behaviour and social interaction ranging from difficulty in maintaining appropriate behaviour in play to a lack of interest in others [8,9].

Criterion B of the DSM is as follows: (B) Restrictive and repetitive patterns of behaviour, interests or activities, manifested by two or more of the following: (B1) stereotyped or repetitive movements, use of objects or speech; (B2) insistence on monotony, excessive inflexibility of routines or ritualised patterns of verbal or non-verbal behaviour; (B3) highly restricted and fixed interests that are not typical in intensity or focus of interest; (B4) hyper- or hypo-reactivity to sensory stimuli or unusual interest in sensory aspects of the environment [10].

Children presenting deficit characteristics in restrictive and repetitive pattens of behaviour demonstrate responses that indicate restricted or repetitive interest. For example, they may not change toys or objects, and they may demonstrate atypical behaviour and impulsivity; self-injurious behaviour; repeating the same question several times; repetitive play; preoccupation with the home environment, school or daily life; an apparent indifference to pain and temperature; adverse responses to specific sounds or textures; excessive sniffing or touching of objects; or visual obsession with light or movement [10].

In addition, children with ASD are classified according to their level of functioning, i.e., grade three if they need very significant help, grade two if they need significant help and grade one if they need help; this diagnosis is established based on developmental and clinical observations of behaviour [9,11].

The severity of the clinical features of ASD may vary from subject to subject, but these features result in significant social, school and occupational underachievement [12]. Manifestations begin in infancy or early childhood, and the influence of socio-cultural factors may have an impact within the spectrum [13].

The Diagnostic and Statistical Manual of Mental Disorders (DSM-5) and its revised screening criteria for ASD aim to improve diagnostic accuracy and consistency, while emphasising the relationship of ASD with sensory processing disorder [6].

Before the DSM-5 issued its diagnostic criteria for ASD with respect to hyper- or hypo-reactivity, the aetiological implications of sensory patterns, and their relevance for assessment and intervention practices, had been reported [14]. The characteristics of sensory symptoms and the high prevalence of these symptoms in infants with ASD and how they can affect infants’ exploration and interaction with the physical and social environment, as well as difficulties in regulating and organising the intensity of responses to sensory stimuli from their body or the environment, had also been described [15].

Since Ayres (1965) [16] conducted his first studies on sensory perception, different terms have been used to describe sensory integration theory [17], as well as to identify and explain concepts related to assessment and treatment. Ayres (1972) [18] defined sensory integration as the neurological process that organises sensations from one’s own body and the environment, enabling effective use of the body within one’s surroundings. It primarily focuses on the vestibular, proprioceptive and tactile sensory systems. Sensory integration encompasses not only dysfunction and intervention, but also their role in learning efficiency. Thus, the theory of sensory integration comprises three broad postulates. The first describes sensory integration and learning, the second defines sensory integration dysfunction and the third guides intervention [17].

Sensory processing refers to how the central nervous system and the peripheral nervous system manage information from sensory systems, which receive, modulate, integrate and organise sensory stimuli, including behavioural responses to sensory information; all of these are components of sensory processing [19]. Furthermore, sensory processing integrates information received from our sensory systems, and as part of this process, our brain interprets, selects, and organises sensory information to appropriately produce motor, behavioural, emotional and attentional responses [20].

The term sensory processing is akin to sensory integration when referring to the central nervous system’s ability to process sensory information. However, the terms are not interchangeable; sensory processing is more expansive than sensory integration because sensory integration is merely a component of sensory processing [19]. In other words, sensory integration (SI) is the therapeutic treatment of sensory processing disorder, utilising play-based motor activities alongside precise challenges to influence how the child responds to sensations [21].

It is estimated that atypical sensory processing occurs in up to 90% of individuals with ASD, potentially affecting all sensory modalities [22], and that approximately 65–80% of children with ASD may experience differences in sensory reactivity [23]. Some sensory experiences for individuals with ASD can be pleasant or self-soothing, while others can be a source of irritation or discomfort [24].

Moreover, atypical sensory processing often coexists with maladaptive or problematic behaviours in children with ASD, and the presence of ASD increases the risk of experiencing social anxiety. It has been found that individuals with ASD are more prone to developing social anxiety than neurotypical individuals [25]. This relationship is even more pronounced in adults with ASD (50–70%) [26], and it also occurs in childhood and adolescence [27].

The sensory hyper-reactivity that this type of population may present has been observed to correlate with clinically elevated levels of anxiety in paediatric populations both with ASD and without ASD [23]. Research has indicated a bidirectional relationship with sensory reactivity increasing anxiety, which, in turn, increases sensory hyper-reactivity [28]. This may be because sensory hyper-reactivity contributes to avoidance, a maladaptive strategy that can further maintain anxiety [23]. Sensory hyper-reactivity in preschool children with ASD is a risk factor for the development of anxiety disorders [29]. Much of the previous research on the bidirectional relationship between anxiety and sensory hyper-reactivity in ASD has focused on sensory reactivity and the presence of anxiety. From a measurement perspective, aggregated scores on broad anxiety measures correlated with the presence of sensory hyper-reactivity. Two more recent studies investigated the relationship between atypical sensory characteristics and subtypes of anxiety [30,31]. Both studies found a relationship connecting hyper-reactivity with specific phobias and separation anxiety. Research has shown that sensory hyper-reactivity correlates with ritualism and symptoms of obsessive compulsive disorder (OCD) in adults [23]. Other research shows that increased sensory input often precedes or accompanies OCD behaviours, including repetitive behaviours and compulsions [23]. Less is known about the links between sensory reactivity differences and OCD in youth with ASD [31].

According to the nosology of sensory processing disorder proposed by Miller et al. (2007) [32], three patterns with their subtypes are included: the first pattern is sensory modulation disorder with its subtypes (hyper, hypo and sensory seeker); the second pattern corresponds to discrimination disorder; and finally, the third pattern belongs to motor-based disorder with its subtypes (dyspraxia and postural disorder). Additionally, Bundy and Lane (2020) [17] devised a schematic representing the relationship between sensory systems and manifestations of sensory integration dysfunction. From the central column outward, it depicts the processing of sensations in the CNS. Indicators of poor sensory integration and praxis are shown to the right, with behavioural consequences such as low self-efficacy and self-esteem, avoidance of participating in motor activities, poor fine motor skills and visuomotor coordination, and poor organisation and sensory seeking. To the left, the schematic shows indicators of modulation dysfunction, with atypical responses evident in sensory challenges related to attention, regulation, affect, and activity; withdrawal from and avoidance of sensory experiences; sensory seeking; and low self-efficacy and self-esteem.

The purpose of this systematic review is to examine the correlation of sensory processing disorder in children with ASD, and intervention with sensory integration as part of therapeutic treatment in children with ASD.

## 2. Method

A review was conducted in accordance with the guidelines and standards for systematic reviews (PRISMA) [33], in which relevant research regarding sensory integration intervention in children ASD was identified, selected and critically evaluated. A search strategy was developed using the academic search engines PubMed, Scielo, Eric, Dialnet, Springer, Base Search and Google Scholar. For this search, controlled vocabulary in both English and Spanish was employed (intervention with sensory integration in infants with ASD spectrum disorder, ASD spectrum disorder and sensory integration, sensory processing disorder in ASD), and to enhance search precision, the Boolean operators AND and OR were used. Finally, the results were restricted to studies published in English and Spanish from 2013 to 2023, yielding 16 articles meeting the inclusion criteria.

### 2.1. Inclusion and Exclusion Criteria

To define the eligibility criteria, the PICO strategy (Population, Intervention, Comparison, and Outcome) was employed [34], and inclusion and exclusion criteria were identified prior to proceeding with the article review. The inclusion criteria considered articles from the period ranging from 2013 to 2023. Systematic reviews and peer-reviewed articles pertaining to sensory integration intervention in children, with an emphasis on ASD, were included, provided they were published in English or Spanish. Conversely, the exclusion criteria involved articles unrelated to childhood, along with book chapters, articles under review, reports, theses, dissertations, proceedings, or works focusing solely on sensory integration, ASD, or sensory processing disorder.

### 2.2. Data Sources and Search Strategies

**Figure 1:**
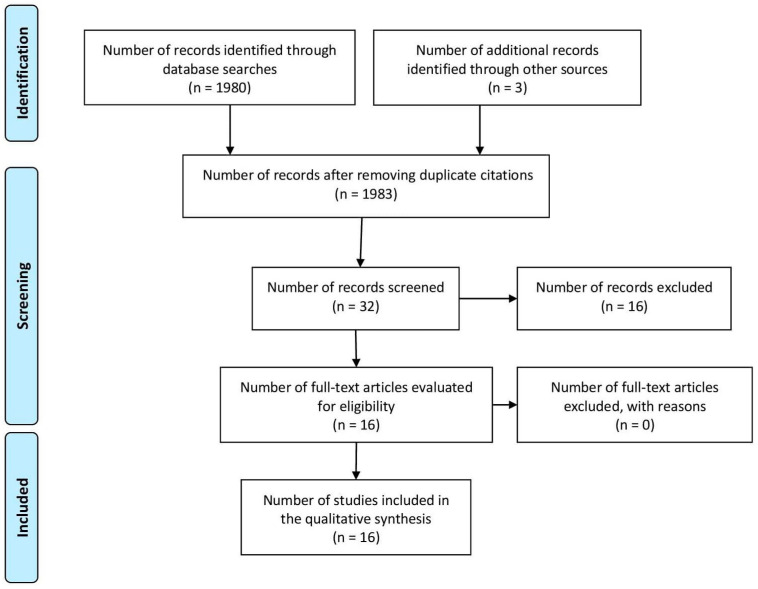
PRISMA diagram.

The PRISMA flowchart in Figure 1 outlines the article selection process. The classification search yielded a large number of results; initially, 1983 articles were identified, but this was reduced to 16 through a rigorous selection process that went through several stages. First, specific inclusion and exclusion criteria were used, and the time span of publication was from 2013 to 2023. In the review stage, duplicate articles and those that did not meet the criteria of the systematic review were detected and deleted. In addition, certain articles were excluded because they were not directly relevant to the research regarding the relationship of sensory integration and ASD. In the review stage, duplicate articles were detected and deleted, and certain articles that were not directly relevant to the research regarding the relationship of sensory integration and ASD were excluded, which reduced our sample to a total of 32, of which *n* = 16 were excluded due to being outside the context of intervention with sensory integration in infants with ASD. Finally, 16 articles met the inclusion criteria and were selected for systematic review.

To facilitate the analysis of the 16 selected articles, a table (Table 2) was prepared including the following: (a) author identifiers, (b) year, (c) study, (d) methodological design of the study and (e) results.

## 3. Results

According to the analysis of the results described below (see Table 2), there are systematic reviews that link sensory integration with ASD, such as the one conducted by [35], which examined the literature related to the effectiveness of Ayres Sensory Integration (ASI) and sensory interventions [18] to improve the daily activities of infants with ASD. This study found moderate evidence suggesting that intensive, individualised ASI clinical intervention can improve functional outcomes in ASD. Three randomized controlled trials with a retrospective analysis and an ABA design concluded that there was moderate evidence of improvement in behaviour and a reduction in caregiver assistance in self-care activities [36]. Another study used the standards for evidence-based practices in special education from the Council for Exceptional Children in order to evaluate the effectiveness of sensory integration intervention for children with ASD, leading them to determine that Ayres Sensory Integration can be considered an evidence-based practice for children with ASD aged 4 to 12 years [37].

In addition to systematic reviews, there are specific research studies, detailed below in chronological order. One of the outstanding studies is a pilot study that investigated the effectiveness of sensory integration in children with high-functioning ASD. A total of 20 children were involved in the research, 8 of whom underwent personalised sensory integration treatment, and the other 12 of whom attended group therapy sessions. The findings indicate that sensory integration may be useful in improving motor, visual and visual–motor skills in preschool children with ASD, suggesting that occupational therapists and experts in the field should consider sensory integration as a valid alternative for the care of this age group [44].

In two reviewed studies, the first assessed gross and fine motor skills in 34 children with ASD (24 boys and 10 girls) before and after a sensory integration programme [50], and the second investigated the relationship between social participation and praxis and sensory integration in 89 children with ASD [45]. These studies underline the importance of sensory integration in improving motor skills and promoting social participation in children with ASD, supporting its consideration as a key component in therapeutic approaches for this group.

Another study looked at the effects of sensory integration therapy on the physical and motor activity of children with ASD. This descriptive study included 20 students from early childhood and primary education diagnosed with ASD, who received a 12-session sensory integration therapy intervention. The authors concluded that sensory integration treatment improved motor ability as well as basic and balance skills [49].

The study by Kashefimehr et al. (2018) [39] examined how sensory integration therapy impacts different areas of occupational performance by using a design that included an intervention and a control group. It was noted in the results that the intervention group showed significant improvements in all domains assessed by the short child occupational profile [39]. In another aspect, Padmanabha et al. (2019) [48] conducted a study examining a home-based sensory intervention for children with ASD, in which two groups were compared: one that received home-based sensory interventions by parents or caregivers along with standard therapy, and another group that only received standard therapy involving speech therapy and applied behaviour analysis [48]. These studies highlight the effectiveness of sensory integration, whether in clinical or home settings, and support its incorporation as a beneficial therapeutic alternative to enhance the performance and health of children with ASD.

In a study of 108 children with ASD who were divided into two groups, a sensory integration intervention group (Group A, *n* = 54) and a control group (Group B, *n* = 54), the members of the two groups were evaluated before and after treatment. Significant differences were observed after the intervention, and it was concluded that sensory integration intervention is of great value for development and intervention in programmes for children with ASD [40].

In a study to evaluate the effects of sensory integration therapy on selected physical skills in children with ASD [46], physical skills and their correlation with sensory integration therapy were evaluated in 15 boys and 5 girls. The results show that sensory integration therapy contributed to an improvement in motor, sensory, cognitive, emotional, communicative and social development in those receiving the intervention, as well as improving physical skills.

A case study involving two children and their caregivers [47] included an initial assessment followed by a six-month intervention, with a subsequent follow-up assessment. Another study [43] investigated how therapists use sensory integration therapy with children with ASD by examining the number and type of activities conducted. A coding scheme was generated in order to detect particular elements of the intervention, using recordings of nine children with ASD to analyse the performance achieved [43]. The findings indicate that sensory interactions and engagement between children and therapists are essential elements in sensory integration therapy, highlighting the importance of sensory intervention in both enhancing sensory processing and strengthening the therapeutic connection. This suggests that it has the potential to be an effective approach in the treatment of children with ASD.

Communication, socialisation and daily living skills improved significantly after sensory integration intervention in a non-randomised controlled trial of 72 infants with ASD [38], while no clinical benefit was demonstrated in a randomised controlled trial [42].

Examining a sensory integration intervention to address play in infants with ASD [41], a non-concurrent multiple baseline design was applied with three children with ASD diagnoses aged 5 and 6 years old. All three participants demonstrated changes in the frequency of specific types of play. If confirmed in future studies, sensory integration intervention could become an evidence-based treatment for improving play.

## 4. Discussion

This systematic review reveals several approaches in which the sensory integration reference model can be applied for the therapeutic treatment of children with ASD.

The results demonstrate that applying sensory integration can significantly improve the behaviour and daily performance of children with ASD. In addition, this structured intervention has shown improvement in communication, socialisation and daily living skills [38].

Consistency in effectiveness has been shown in the articles reviewed, as corroborated by systematic reviews concluding that Ayres sensory integration is an evidence-based practice [37] (Schoen et al., 2019). Of particular note is the study whose findings suggest that integration intervention can significantly modify children’s play patterns [41].

Despite these positive findings, it is necessary to note that the heterogeneity of the interventions and the different methodologies of the studies limit our ability to generalise the effects of the interventions. Furthermore, a lack of standardisation of methods suggests that more rigorous studies are needed to confirm these results and develop clearer applications for practice in other non-clinical settings.

The results of this review support the efficacy of sensory integration in relation to previous research proposed by Watling and Hauer (2015) [35] and Schoen et al. (2019) [37]. In this regard, the results are consistent with the idea that the sensory integration approach is beneficial in improving the adaptive responses of minors with ASD.

In addition, the results of this systematic review have notable implications that impact clinical practice, and support the existing sensory integration theory in the treatment of children with ASD. They also emphasise the diversity of children with ASD, which is why the therapeutic approach needs to consider each child individually.

The results of this systematic review suggest several key contributions for occupational therapists who work with children with ASD and apply the sensory integration model in their treatment. Occupational therapists who apply this model must base their work on approaches that have demonstrated efficacy, and these interventions must be adapted to the individual needs of each child, because the response to stimuli may vary from one subject to another. Within sensory integration treatment, a wide range of adaptive responses that will benefit children with ASD should be considered, for example, including strategies that involve communication responses, social skills, psychomotor skills, etc. It is important for professionals to undertake continuous learning and undergo updated training according to the latest evidence-based methods and techniques in order to improve their skills and the effectiveness of the intervention, and guarantee the expected results.

To strengthen these findings on the efficacy of sensory integration, it is crucial to explore several areas of further research that encompass different cultural backgrounds in order to explore how treatment effects vary in different populations and to tailor interventions according to the specific needs of each group. Future studies should evaluate the persistence of sensory integration effects over the long term, including the impact of adaptive responses in school, social and family environments.

The results from this review show significant responses for the ASD spectrum disorder community who, thanks to this intervention, tend to experience significantly improvements in their quality of life, in addition to how they manage their sensory processing. This leads to greater participation in day-to-day activities that promotes better adaptation in educational and social environments. And thanks to the intervention, their families can also be included in the process. This research provides families with valuable information about which interventions are supported by scientific evidence, which will help them make informed decisions about the best treatment for their children.

This systematic review makes an important contribution, as it performs a comprehensive evaluation of interventions in a given time. This contributes to the literature and provides a clear picture of which treatments are evidence-based. In addition, it provides a critical analysis of the practices and their results, allowing professionals to select and apply treatments with a solid research base.

## 5. Conclusions

In conclusion, the results of this article highlight the importance of sensory integration intervention in the treatment of children with ASD. The research reviewed demonstrates that sensory integration therapy can have a positive effect in several areas in children diagnosed with ASD, including motor, cognitive, social and communication skills, as well as a significant improvement in quality of life and daily functioning in children who receive this intervention. Furthermore, sensory integration intervention for children with ASD is carried out by certified and qualified professionals, who meet the criteria for this to be considered an evidence-based practice. The results also highlight the importance of considering sensory integration intervention as an integral part of treatment programmes for children with ASD, complementing other therapeutic and educational interventions to comprehensively address these children’s needs. However, the research also points to the need to address differences in outcomes, as well as the lack of success in some studies on the clinical benefits of sensory integration therapy. This highlights the importance of continuing to investigate and critically evaluate the effectiveness and applicability of this intervention in the ASD population.

In addition, the information obtained through this research shows a relationship between sensory processing and anxiety in children with ASD. However, the articles reviewed do not establish whether there was an evaluation process on the impact of anxiety prior to the sensory integration approach. Finally, most of these studies were conducted from a clinical perspective, which makes new research necessary on intervention with sensory integration from other ecological environments that are part of the daily lives of children with ASD, such as the home and school.

## Figures and Tables

**Table 1:** Evolution of the ASD diagnosis in the DSM.

Evolution of the DSM			
1980 DSM III	1980 DSM III-R	1994 DSM IV	2013 DSM V
Introduction of the diagnosis of autism disorder.	Subtypes such as Asperger’s syndrome are added.	The term autism spectrum disorders (ASD) becomes established.	Unification of subtypes under the term autism spectrum disorder and elimination of terms such as Asperger’s syndrome.

**Table 2:** Analysis of articles.

Authors	Year	Study	Language	Method	Results
Renee Watling; Sarah Hauer [35]	2015	Effectiveness of Ayres Sensory Integration^®^ and Sensory-Based Interventions for People With Autism Spectrum Disorder: A Systematic Review.	English	Literature published from January 2006 to April 2013 related to the effectiveness of Ayres Sensory Integration^®^ (ASI) and sensory-based interventions (SBI) was reviewed.	Moderate evidence was found to support the use of ASI. The results of the sensory methods were mixed.
Roseann C. Schaaf; Rachel L. Dumont; Marian Arbesman; Teresa A. May-Benson [36]	2018	Efficacy of Occupational Therapy Using Ayres Sensory Integration^®^: A Systematic Review.	English	The review included 3 randomised controlled trials, 1 retrospective analysis and 1 single-subject ABA design, published between 2007 and 2015, that studied children with ASD.	Moderate evidence supported improvements in impairment-level outcomes of improvement in autistic behaviours and skills-based outcomes of reduced caregiver assistance with self-care activities.
Schoen SA, Lane SJ, Mailloux Z, May-Benson T, Parham LD, Smith Roley S, Schaaf RC. [37]	2019	Ayres Systematic Review of Sensory Integration for Children with Autism.	English	This study used the Council for Exceptional Children’s standards to review evidence-based practice in special education in three stages.	This study evaluated the effectiveness of sensory integration research from 2006 to 2017, and determined that Ayres Sensory Integration is an evidence-based practice and is frequently requested by parents.
Citra Raditha, Setyo Handryastuti, Hardiono D Pusponegoro, Irawan Mangunatmadja [38]	2023	Positive behavioral effect of sensory integration Intervention in Young Children with Autism Spectrum disorder.	English	Non-randomised controlled trial of assessment using the Vineland Adaptive Behaviour Scale II.	A total of 72 subjects were studied and it was concluded that after the intervention communication, socialisation and daily living skills improved significantly.
Babak Kashefimehr, Hülya Kayihan, Meral Huri [39]	2018	The Effect of Sensory Integration Therapy on Occupational Performance in Children with Autism.	English	Randomised controlled trial where both the control and intervention groups were homogeneous in terms of age, and with a diagnosis of ASD grade 3.	The intervention group showed significantly greater improvement in all domains of the short child occupational profile.
Wenxin Xu, Jiwei Yao, Wenyao Liu [40]	2019	Intervention Effect of Sensory Integration Training on the Behaviors and Quality of Life of Children with Autism.	English	Randomised method. From September 2017 to December 2018, 108 participants were included in the intervention group and control group, with 54 members in each group.	Significant differences were observed after treatment, and it was concluded that the SI intervention had some effect on ASD and is of great value for the development of intervention programmes for children with ASD.
Heather M Kuhaneck, Renee Watling, Tara J Glennon [41]	2023	Ayres Sensory Integration^®^ for Addressing Play in Autistic Children: A Multiple-Baseline Examination.	English	Non-concurrent multiple baseline design across subjects.	The findings suggest that ASI intervention can alter a child’s play patterns.
Elizabeth Randell, Melissa Wright, Sarah Milosevic, David Gillespie, Lucy Brookes-Howell, Monica Busse-Morris, Richard Hastings, Wakunyambo Maboshe, Rhys Williams-Thomas, Laura Mills, Renee Romeo, Nahel Yaziji, Anne Marie McKigney, Alka Ahuja, Gemma Warren, Eleni Glarou, Sue Delport, Rachel McNamara [42]	2022	Sensory Integration Therapy for Children with Autism and Sensory Processing Difficulties.	English	A parallel-group randomised controlled trial, incorporating an internal pilot and process evaluation. The trial included 138 participants, *n* = 69 per group.	The clinical effectiveness and cost-effectiveness of sensory integration therapy for children with ASD and sensory difficulties were tested.
Cristin M Holland, Erna I Blanche, Barbara L Thompson [43]	2021	Quantifying Therapists’ Activities during Sensory Integration Treatment for Young Children with Autism.	English	A coding scheme that identifies specific aspects of sensory integration treatment was developed and used to analyse 34 videos of 9 children with ASD.	Quantification of therapists’ actions can provide information about moment-to-moment decision making and therapist–child relationships during the daily practice of sensory integration treatment.
Ryoichiro Iwanaga, Sumihisa Honda, Hideyuki Nakane, Koji Tanaka, Haruka Toeda, Goro Tanaka [44]	2014	Pilot study: efficacy of sensory integration Therapy for Japanese Children with High-Functioning AutismSspectrum Disorder.	English	The subjects were 20 children with TEA with IQs above 70 selected from previously collected data.	Sensory integration therapy was most effective for motor coordination skills, nonverbal cognitive skills and combined sensorimotor and cognitive skills in children with ASD.
Susanne Smith Roley, Zoe Mailloux, L. Diane Parham, Roseann C. Schaaf, Christianne Joy Lane, Sharon Cermak [45]	2015	Sensory Integration and Praxis Patterns in Children With Autis.	English	Tests of sensory integration and practice (SIPT) and sensory processing measures (SPM) were conducted on clinical records of children with ASD aged 4–11 years (*n* = 89).	Children with ASD characteristically show strengths in visopraxia and difficulties with somatopraxis and vestibular functions, which appear to greatly affect participation.
Włodzisław Kuliński, Adela Nowicka [46]	2020	Effects of Sensory Integration Therapy on Selected Fitness Skills in Autistic Children.	English	This study evaluated a group of 20 children (15 boys and 5 girls) aged 3–10 years. All children were diagnosed with ASD and underwent 2 years of therapy. The study assessed physical skills and their correlations with the sensory integration therapy used with the children.	Sensory integration therapy contributed to improved motor, sensory, cognitive, emotional, communicative and social development in the study patients.
Francielly Caroline Silva Costa and Luzia Iara Pfeifer [47]	2021	Sensory Integration Intervention for Children with Autistic Spectrum Disorder.	Spanish	This was a case study that was exploratory and qualitative in nature.	It was confirmed that the results of this study corroborate those found in the literature, i.e., based on sensory integration, interventions favour the improvement of various aspects of sensory processing and improved functional performance.
Hansashree Padmanabha, Pratibha Singhi, Jitendra Kumar Sahu, Prahbhjot Malhi [48]	2018	Home-based Sensory Interventions in Children with Autism Spectrum Disorder: A Randomized Controlled Trial.	English	This was a 12-week pilot, parallel-group, randomised controlled trial.	The findings suggest that sensory interventions in the home are feasible in a developing country and are suggested to have a beneficial role in ASD.
Leyla Sadat Karimi, Seyed Ebrahim Hosseini, Farzaneh Manzari- Tavakoli [49]	2017	The Effects of Sensory Integration Therapy on Physical and Motor Activity of Children with Autism Spectrum Disorders in Shiraz.	English	This descriptive study was carried out with 20 students selected in the first, second and third grades aiming to diagnose ASD in an academic setting.	Integrative therapy improves daily, organised and motor activity as well as basic and balance skills in children with ASD and may require longer treatments to improve kinesthetic skills.
Amel E. Abdel Karim, Amira H. Mohammed [50]	2015	Effectiveness of Sensory Integration Program in Motor Skills in Children with ASD.	English	Thirty-four children of both sexes with ASD (24 males and 13 females) participated in this study. They ranged in age from 40 to 65 months. The children were assessed before and after SI treatment using the Peabody Developmental Motor Scale (PDMS-2).	This study revealed a significant improvement in gross and fine motor skills. Sensory integration therapy is effective in the treatment of autistic children as it helps them to become more independent and participate in everyday activities.

## Data Availability

The data presented in this study are available on request from the corresponding author. The data are not publicly available due to ethical reasons and for confidentiality.
